# Electrochemical Skin Conductance May Be Used to Screen for Diabetic Cardiac Autonomic Neuropathy in a Chinese Population with Diabetes

**DOI:** 10.1155/2017/8289740

**Published:** 2017-02-09

**Authors:** Tianyi He, Chuan Wang, Anju Zuo, Pan Liu, Ruxing Zhao, Wenjuan Li, Li Chen, Xinguo Hou

**Affiliations:** ^1^Department of Endocrinology, Qilu Hospital, Shandong University, Jinan, Shandong 250012, China; ^2^Institute of Endocrinology and Metabolism, Shandong University, Jinan, Shandong 250012, China

## Abstract

*Aims*. This study aimed to assess whether the electrochemical skin conductance (ESC) could be used to screen for diabetic cardiac autonomic neuropathy (DCAN) in a Chinese population with diabetes.* Methods*. We recruited 75 patients with type 2 diabetes mellitus (T2DM) and 45 controls without diabetes. DCAN was diagnosed by the cardiovascular autonomic reflex tests (CARTs) as gold standard. In all subjects ESCs of hands and feet were also detected by SUDOSCAN™ as a new screening method. The efficacy was assessed by receiver operating characteristic (ROC) curve analysis.* Results*. The ESCs of both hands and feet were significantly lower in T2DM patients with DCAN than those without DCAN (67.33 ± 15.37 versus 78.03 ± 13.73, *P* = 0.002, and 57.77 ± 20.99 versus 75.03 ± 11.41, *P* < 0.001). The ROC curve analysis showed the areas under the ROC curve were both 0.75 for ESCs of hands and feet in screening DCAN. And the optimal cut-off values of ESCs, sensitivities, and specificities were 76 *μ*S, 76.7%, and 75.6% for hands and 75 *μ*S, 80.0%, and 60.0% for feet, respectively.* Conclusions*. ESC measurement is a reliable and feasible method to screen DCAN in the Chinese population with diabetes before further diagnosis with CARTs.

## 1. Introduction

The prevalence of diabetes is increasing rapidly worldwide [1], especially in China [2]. 60% to 70% of patients with type 2 diabetes mellitus (T2DM) suffer from diabetic neuropathy, including diabetic cardiovascular autonomic neuropathy (DCAN), which can lead to increased risk of cardiovascular mortality [3]. The American Diabetes Association (ADA) has recommended that physicians screen for DCAN at the time of diagnosis for patients with T2DM and within 5 years of diagnosis for patients with type 1 diabetes mellitus (T1DM) [4]. Unfortunately, the significance of DCAN has generally been overlooked in current clinical diagnostic and treatment routines, and physicians have had no practical point-of-care tool available for the detection of subclinical DCAN [5]. The battery of cardiovascular autonomic reflex tests known as CARTs is widely accepted as the gold standard to screen for DCAN [6, 7]. However, CARTs have significant disadvantages [7] such as cumbersome administration, subjective criteria, time-consuming analysis, and weak repeatability, which make them ill-suited for annual DCAN screening. Sudomotor dysfunction, characterized by sweating deficiency due to loss of small sympathetic nerve fibers, has been shown to develop early in the course of diabetes. Electrochemical skin conductance (ESC) measured by the SUDOSCAN technology (Impeto Medical, Paris, France) is a new index to detect sudomotor dysfunction early and rapidly; it has been used in previous studies to screen for prediabetes and diabetes, as well as in the detection of diabetic microvascular complications and DCAN [8–14]. Since the assessment of sudomotor function has been proposed to evaluate autonomic disturbances [15], this study aimed to explore whether this new ESC index could reliably screen for DCAN in the Chinese population with diabetes and reduce the number of subjects tested with CARTs.

## 2. Materials and Methods

### 2.1. Study Population

A total of 75 patients with T2DM and 45 nondiabetic controls were recruited at Qilu Hospital of Shandong University from March to August 2014. All diabetes patients were inpatients. To match the age and sex of the two groups, we chose as controls similarly aged spouses or relatives of the T2DM patients or members of the hospital cleaning staff most of whom had never taken drugs for chronic disease before. We excluded those with diabetes, hypertension, coronary heart disease, and so forth. Diabetes mellitus was defined according to the 2013 ADA diagnostic criteria [4]. Study exclusion criteria were as follows: presence of (or history of) acute myocardial infarction, cerebral hemorrhage, severe hypertension, and implanted cardiac pacemaker; severe thyroid, hepatic, or renal disease; retinal proliferative lesions or retinal hemorrhage; taking any of the following medications within one month of study enrollment: digoxin, *β*-blockers, and antidepressants; long-term consumption of coffee, tea, alcohol, or other caffeinated drinks. The study was conducted in accordance with the principles of the Helsinki Declaration and approved by the Qilu Hospital research ethics committee. All subjects provided signed informed consent.

### 2.2. Data Collection and Clinical Evaluation

Basic information was collected from all subjects by professional physicians, including medical history, age, and gender. A clinical examination was administered to record height, weight, waist circumference, and blood pressure. After fasting for at least 8 hours, venous blood was collected from both the T2DM and control groups for measurement of fasting plasma glucose (FPG, by automatic biochemical analyzer 400, Toshiba, Japan, 3.9–6.1 mmol/L), fasting C-peptide (FC-P by immune chemiluminescence apparatus, BAYER CENTAUR, 0.81–3.85 ng/mL), fasting insulin (FINS, by immune chemiluminescence apparatus, BAYER CENTAUR, 5–10 *μ*IU/mL), and glycated hemoglobin A1c (HbA1c, by high pressure liquid chromatograph, VARIANT II, Bio-Rad, 4–6%) in the endocrinology laboratory of Qilu Hospital.

Cardiovascular autonomic reflex tests were used as the gold standard clinical testing method [7]. The whole process was conducted by ECG according to Ewing et al. [16] and included heart rate (HR), response to deep breathing (the difference between the maximum and minimum heart rates during each deep expiration and inspiration at 6 breaths per minute), Valsalva maneuver (the ratio of the longest R-R interval shortly after Valsalva maneuver to the shortest R-R interval during Valsalva maneuver), heart rate response to standing (30 : 15 ratio, the ratio of the R-R intervals of the 30th beat to the 15th beat cycle after standing up unaided), and postural blood pressure change (the difference in systolic blood pressure change between lying down and standing up after 2 min). Diagnostic criteria and staging of DCAN are still being debated. The Toronto Diabetic Neuropathy Expert Group [7, 17] suggests that at least two abnormal HR tests are required for a definite or confirmed diagnosis of cardiovascular autonomic neuropathy. However, this grading system fails to consider the relative effect of each CART; therefore, in the present study, we selected the other recommended scoring system (Table 1) [16] and use the total score to define the severity of cardiovascular autonomic dysfunction. According to the scoring system a normal result scores 0 points, a borderline result 1 point, and an abnormal result 2 points. The total scores are calculated by adding individual points. Severity groups of DCAN were divided according to the total score: 0-1 point was defined as no-DCAN (no-DCAN group); 2 to 3 points denoted early-DCAN (early-DCAN group); and 4 to 8 points confirmed definite-DCAN (definite-DCAN group).

ESC was measured using the SUDOSCAN device (Impeto Medical, Paris, France). Participants were asked to place their bare hands and feet on stainless steel electrode plates. The device applies incremental low direct current (DC) voltage potential (less than 4 V) to the plates during a 2-minute testing period. Electrochemical skin conductance (ESC), derived from the sweat chloride ion current produced in response to the applied voltages, is automatically calculated by the equipment for each hand and foot. The test is painless, noninvasive, portable, and very simple to operate.

### 2.3. Statistical Analysis

The data are presented as mean ± Standard Deviation (SD). Independent sample *t* tests were used to compare two groups while multiple groups using one-way ANOVA (analysis of variance). Chi-square tests were used to compare categorical variables between groups. Receiver operating characteristic (ROC) curve was used to evaluate the sensitivity and specificity of the diagnostic evaluation methods. Significance was defined as a two-tailed *P* < 0.05. Statistical procedures were performed with the statistical package SPSS 17.0.

## 3. Results

### 3.1. Clinical Features of T2DM Patients and Controls

A total of 75 patients with T2DM and 45 nondiabetic controls were included in the study. As shown in Table 2, waist circumference, diastolic blood pressure, and relevant indicators of glucose metabolism in the T2DM patients were significantly higher than in the controls, while age, BMI, and systolic blood pressure were not different between the two groups. The means of hands ESC and feet ESC, the indicators of sudomotor function, were significantly lower in T2DM patients than in controls (hands ESC, *P* = 0.046; feet ESC, *P* = 0.025). The total CARTs score was higher in T2DM patients than in controls. In 45 Chinese controls, the mean hands ESC was 78.36 ± 9.74 *μ*S and the mean feet ESC was 73.36 ± 9.78 *μ*S.

### 3.2. Ratio of DCAN in T2DM Patients and Controls

**Figure 1:**
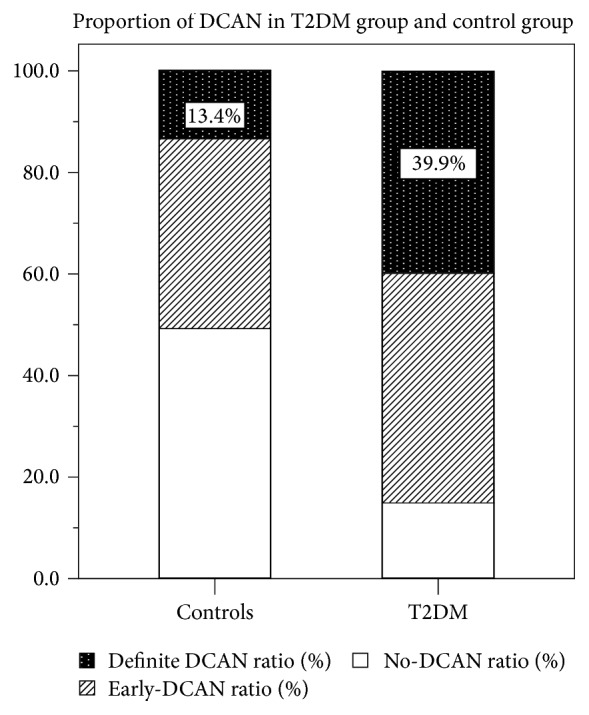
Proportion of confirmed diabetic cardiovascular autonomic neuropathy (definite-DCAN), early stage of diabetic cardiovascular autonomic neuropathy (early-DCAN), and no diabetic cardiovascular autonomic neuropathy (no-DCAN) in patients with type 2 diabetes mellitus (T2DM) and controls.

Based on CARTs total scores, 39.9% (30/75) of T2DM patients were diagnosed with DCAN as shown in Figure 1. It should be noted that 13.4% (6/45) of controls were also diagnosed with nondiabetic cardiac autonomic neuropathy. Certainly, however, the ratio of DCAN was significantly higher in T2DM patients than in controls (*P* = 0.002). Otherwise, the proportion of the no-DCAN group in controls was 48.9% (22/45), which was much higher than the 14.7% (11/75) found in T2DM groups.

### 3.3. Comparison of Electrochemical Skin Conductance (ESC) in DCAN and No-DCAN Subjects

**Figure 2:**
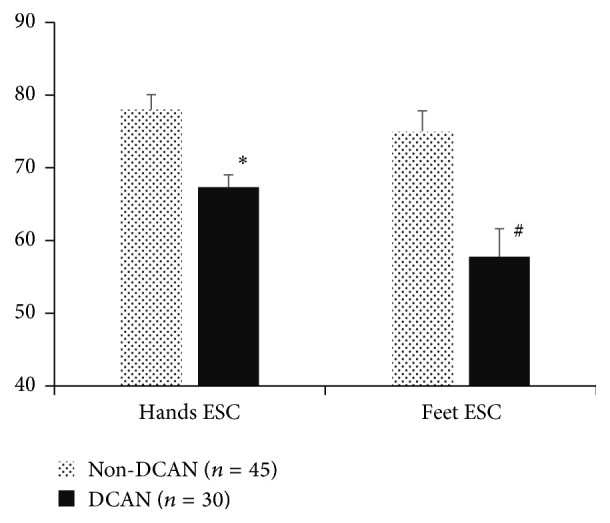
Feet and hands electrochemical skin conductance (ESC) in subjects with cardiovascular autonomic neuropathy (DCAN) and subjects without diabetic cardiovascular autonomic neuropathy (no-DCAN). Data are mean ± SD values. ^*∗*^*P* = 0.002. ^#^*P* < 0.001.

As shown in Figure 2, both hands and feet ESC in T2DM patients with DCAN were significantly lower than in T2DM patients without DCAN (67.33 ± 15.37 versus 78.03 ± 13.73, *P* = 0.002, and 57.77 ± 20.99 versus 75.03 ± 11.41, *P* < 0.001). Using CARTs total score as the standard, the T2DM patients were divided into 30 cases of DCAN, 34 cases of early-DCAN, and 11 no-DCAN patients (Table 3). Compared with no-DCAN patients, patients with DCAN and early-DCAN demonstrated lower ESC, which is positively correlated with the severity.

### 3.4. Diagnostic Efficiency of ESC for Screening DCAN

**Figure 3:**
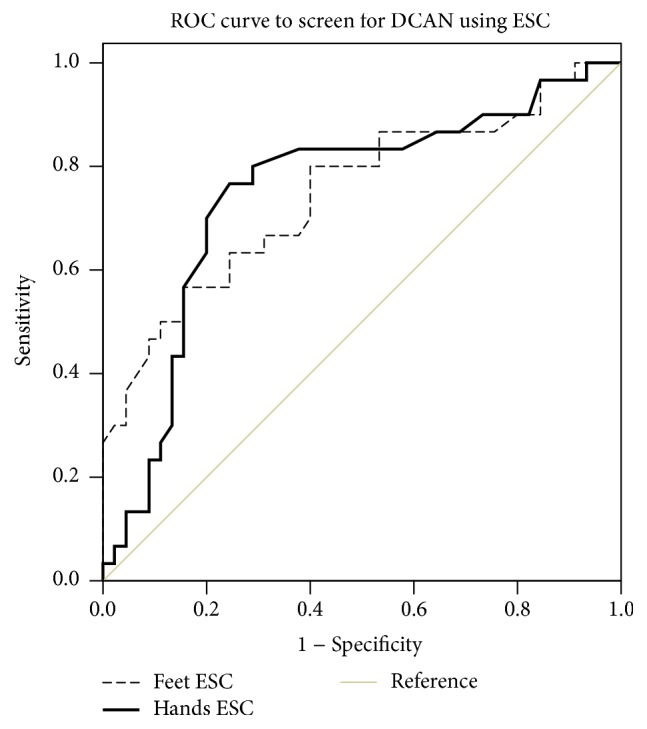
Receiver operating characteristic (ROC) curve of mean hands electrochemical skin conductance (ESC) and mean feet ESC to screen for diabetic cardiovascular autonomic neuropathy (DCAN) in diabetes group, using the cardiovascular autonomic reflex tests (CARTs) total score as the criteria to diagnose DCAN. The areas under the ROC curve (AUC) of mean hands ESC (black line) and feet ESC (dashed line) to predict DCAN were 0.750 and 0.747, respectively. *P* value < 0.01.

Using CARTs total score as the standard, we evaluated the diagnostic efficiency of ESC for screening DCAN in patients with T2DM. The areas under the ROC curve (AUC) of mean hands ESC and mean feet ESC were 0.750 (95% CI: 0.631~0.869) and 0.747 (95% CI: 0.630~0.865) separately (Figure 3). The accuracy of ESC to screen for DCAN is shown in Table 4. Corresponding to the highest Youden index (feet ESC, 0.400; hands ESC, 0.522), the optimal cut-off values of mean hands ESC and mean feet ESC were 75.76 *μ*S and 75.19 *μ*S, respectively. The sensitivity and specificity for optimal mean hands ESC cut-off value were 76.7% and 75.6%, respectively, while the sensitivity and specificity for optimal mean feet ESC cut-off value were 80.0% and 60.0%, respectively.

## 4. Discussions

DCAN is one of the common and chronic complications of diabetic neuropathy (DN). In the early phases it is characterized by an insidious onset, manifesting as resting tachycardia, exercise intolerance, and orthostatic hypotension [12]. In the late phase, a meta-analysis of DCAN and mortality showed that DCAN was strongly associated with higher mortality risk owing to acute painless myocardial infarction [18, 19]. Even so, current screening methods for DCAN are neglected, and only resting heart rate is monitored in regular clinical practice [20]. DCAN was detected in only 7% of T1DM and T2DM at the time of diagnosis. In this study, we explored how the assessment of sudomotor function by measuring hands and feet ESC could be a helpful and practicable tool to screen for DCAN in Chinese subjects in clinical practice.

Traditionally, CARTs are the gold standard tests for diagnosing cardiovascular autonomic neuropathy and are recommended not only by the American Diabetes Association but also by the Cardiovascular Autonomic Neuropathy Subcommittee of the Toronto Consensus Panel [7]. CARTs were put forward by Ewing et al. in 1985 [16] and comprise five tests. In many large clinical studies [3], three to five CARTs tests are used as diagnostic criteria. The sustained handgrip test is less commonly selected than others, for example. In some studies the Valsalva maneuver is excluded for reasons of patient safety [12]. On the subject of patient safety we excluded diabetes with retinal proliferative lesions or retinal hemorrhage during enrollment as a safety precaution. In our study we used four CARTs tests (Table 1) as the diagnostic standard for DCAN. Owing to a few disadvantages such as tediousness and time-consuming operation [12], CARTs are not usually performed in everyday clinical practice until later, more severe typical manifestations of dysautonomia arise such as orthostatic hypotension. Currently, screening rates for and awareness of DCAN are relatively low. Given DCAN's association with high risk of mortality discussed above, we think it is advisable to find an easier method to screen for DCAN. According to this study's screening results, the number of patients who need to be diagnosed by CARTs should be reduced significantly, especially in countries with increasingly large diabetic populations such as China.

Apart from quantitative sudomotor axon reflex testing (QSART), we have discussed how sudomotor function can also be measured by electrochemical skin conductance (ESC) using the SUDOSCAN device, which applies low amplitude voltages (less than 4 V) to the palms and soles and monitors the variability of the ionic flow (Cl^−^) through sweat glands [8, 21]. A recent study of a healthy Chinese population (*n* = 120) [22] found mean hands ESC values of 61.2 ± 15.5 *μ*S and mean feet ESC values of 69.1 ± 16.8 *μ*S. In our study, both feet and hands ESC in controls were higher than those measured in the former study (78.36 ± 9.74 *μ*S and 73.36 ± 9.78 *μ*S, resp.). Sudomotor dysfunction has been observed in both prediabetes and diabetes and is closely linked to impaired epidermal C-nerve fibers, which are themselves associated with chronic high glucose [23]. We verified that both feet and hands ESC in diabetic subjects were lower than in controls (Table 2). Furthermore, ESC scores in those patients with DCAN were much lower than in those without DCAN (Figure 2). Mean hands ESC and mean feet ESC may be independent predictors of DCAN, as confirmed by ROC curve analysis (Figure 3). Similarly, Casellini et al. evaluated the relationship of hands and feet ESC to diabetic peripheral neuropathy including autonomic function, with a resultant AUC of 0.86 and 0.88, respectively [24]. Selvarajah et al. in the UK and Yajnik et al. in India [14, 25] have also conducted similar international studies.

Compared to the results of CARTs, the ESC report is quite easy to understand. Quantitative results and the lack of time-consuming and complex data analysis are both advantages of the ESC test for the clinical physician. At present, related research concerning the sensitivity and reliability of ESC for DCAN risk screening is very limited. Moreover, research data in Chinese populations are also scarce. We therefore recommend further exploration of the application of the ESC test in screening for DCAN.

Our study has a few limitations: (i) the sample size was relatively small and limited to the Shandong province of China, (ii) the subjects we studied underwent treatment with different types of antidiabetic drugs, meaning the results may not be free of the effect of therapeutic medicine, and (iii) subjects had no other evaluation of small fiber neuropathy.

In conclusion, ESC measurement is reliable and feasible to screen for DCAN among Chinese diabetic patients as a noninvasive, quantitative, and fast method especially in routine clinical practice and large-scale epidemiological surveys before further diagnosis with cardiovascular reflex tests. Further research is needed to confirm the above results and explore new applications of ESC measurement in the management of diabetic neuropathy.

## Figures and Tables

**Table 1:** Components and scoring system for the cardiovascular reflex tests [7, 16].

CARTs	Scores (values)		
	Normal (0 points)	Borderline (1 point)	Abnormal (2 points)
(A) HR response to deep breathing	*⩾*15	11–14	⩽10
(B) Valsalva ratio	*⩾*1.21	1.11–1.20	⩽1.10
(C) HR response to standing (30 : 15 ratio)	*⩾*1.04	1.01–1.03	⩽1.00
(D) Postural blood pressure change	⩽10	11–29	*⩾*30

CARTs, cardiovascular reflex tests.

**Table 2:** Clinical features of T2DM patients and controls.

	T2DM (*n* = 75)	Controls (*n* = 45)	*P* value (<0.05)
Age (yrs)	55.55 ± 14.36	50.80 ± 12.48	0.068
Male (*n*, %)	53.33%	46.67%	0.572
BMI (kg/m^2^)	26.55 ± 6.01	25.21 ± 3.34	0.171
SBP (mmHg)	132.17 ± 18.24	127.27 ± 17.64	0.151
DBP (mmHg)	79.28 ± 13.66	71.98 ± 10.21	0.001^*∗∗*^
Waist circumference (cm)	93.80 ± 13.04	85.08 ± 10.18	<0.001^*∗∗*^
HbA_1C_ (%)	8.89 ± 2.28	5.34 ± 0.36	<0.001^*∗∗*^
FPG (mmol/L)	8.42 ± 2.91	5.50 ± 0.52	<0.001^*∗∗*^
FC-P (ng/mL)	1.55 ± 0.89	1.23 ± 0.52	0.03^*∗*^
FINS (uIU/mL)	14.02 ± 10.37	6.07 ± 2.68	<0.001^*∗∗*^
HOMA-IR	4.87 ± 3.27	1.49 ± 0.68	<0.001^*∗∗*^
CARTs total score	3.23 ± 1.67	2.04 ± 1.58	<0.001^*∗∗*^
Mean hands ESC (*μ*S)	73.75 ± 15.25	78.36 ± 9.74	0.046^*∗*^
Mean feet ESC (*μ*S)	68.13 ± 17.96	73.81 ± 9.34	0.025^*∗*^

Data were mean ± SD for continuous variables and *n* (%) for categorical variables. BMI, body mass index; SBP, systolic blood pressure; DBP, diastolic blood pressure; FPG, fasting plasma glucose; FC-P, fasting C-peptide; FINS, fasting insulin; HOMA-IR, Homeostatic Model Assessment of Insulin Resistance; T2DM, type 2 diabetes mellitus; CARTs, cardiovascular reflex tests; ESC, electrochemical skin conductance; *P* values were for one-way ANOVA or Chi-square tests across the 2 groups. ^*∗*^*P* < 0.05. ^*∗∗*^*P* < 0.001.

**Table 3:** Comparison of different indicators in different severity DCAN.

	T2DM			*F* value	*P* value
	No-DCAN group (*n* = 11)	Early-DCAN group (*n* = 34)	Definite-DCAN group (*n* = 30)		
Age (yrs)	48.00 ± 14.89	56.56 ± 10.82	57.17 ± 17.07	1.834	0.167
Duration of DM (yrs)	7.32 ± 9.35	9.81 ± 7.56	9.03 ± 8.57	0.383	0.683
BMI (kg/m^2^)	26.30 ± 4.32	25.61 ± 4.27	27.70 ± 7.91	0.974	0.382
SBP (mmHg)	131.09 ± 15.12	133.35 ± 21.52	131.23 ± 15.51	0.127	0.881
DBP (mmHg)	75.00 ± 8.33	81.12 ± 15.46	78.77 ± 13.01	0.865	0.425
Waist circumference (cm)	92.36 ± 10.61	92.79 ± 12.37	95.47 ± 14.69	0.406	0.668
Resting Heart rate (bmp)	71.00 ± 7.48	72.62 ± 8.36	78.53 ± 10.40	0.707	0.497
HbA_1C_ (%)	9.05 ± 2.07	8.50 ± 2.21	9.28 ± 2.43	0.944	0.300
FPG (mmol/L)	7.58 ± 3.15	8.11 ± 2.98	8.34 ± 2.75	1.074	0.347
FC-P (ng/mL)	1.87 ± 0.61	1.38 ± 0.90	1.63 ± 0.94	1.510	0.228
FINS (uIU/mL)	12.43 ± 6.63	13.46 ± 4.57	15.25 ± 15.25	0.382	0.684
Mean hands ESC (*μ*S)	79.73 ± 13.12^a*∗*^	77.81 ± 14.11^c*∗*^	67.33 ± 15.37	4.916	0.010^*∗*^
Mean feet ESC (*μ*S)	72.32 ± 17.64^b*∗*^	75.91 ± 8.72^d*∗∗*^	57.77 ± 20.99	10.707	<0.001^*∗∗*^

Data were mean ± SD for continuous variables and *n* (%) for categorical variables. BMI, body mass index; SBP, systolic blood pressure; DBP, diastolic blood pressure; FPG, fasting plasma glucose; FC-P, fasting C-peptide; FINS, fasting insulin; HOMA-IR, Homeostatic Model Assessment of Insulin Resistance; T2DM, type 2 diabetes mellitus; HC, health controls; DCAN, diabetic cardiovascular autonomic neuropathy; no-DCAN, no diabetic cardiovascular autonomic neuropathy; ESC, electrochemical skin conductance; *F* values and *P* values were for one-way ANOVA across the 3 groups. ^a^*P* = 0.029; ^b^*P* = 0.012; ^c^*P* = 0.005; ^d^*P* < 0.001, compared with the diagnosed DCAN group. ^*∗*^*P* < 0.05. ^*∗∗*^*P* < 0.001.

**Table 4:** Diagnostic efficiency of electrochemical skin conductance in the screening of diabetic cardiac autonomic neuropathy.

	Criterion^*∗*^	Sensitivity (%)	Specificity (%)	+PV (%)	−PV (%)	TC (%)
Feet ESC	75.19 *μ*S	80.0	60.0	57.1	81.8	68.0
Hands ESC	75.76 *μ*S	76.7	75.6	67.6	82.9	76.0

ESC, electrochemical skin conductance; +PV, positive predictive value; −PV, negative predictive value; TC, total consistence rate. ^*∗*^Criterion corresponding to the highest Youden index (feet ESC, 0.400; hands ESC, 0.522).
